# Effects of Tree-crop Farming on Land-cover Transitions in a Mosaic Landscape in the Eastern Region of Ghana

**DOI:** 10.1007/s00267-018-1060-3

**Published:** 2018-05-11

**Authors:** Kwabena Asubonteng, Karin Pfeffer, Mirjam Ros-Tonen, Jan Verbesselt, Isa Baud

**Affiliations:** 1 Amsterdam Institute for Social Science Research (AISSR), University of Amsterdam, P.O. Box 15629, 1001 NC Amsterdam, The Netherlands; 2 United Nations University Institute for Natural Resources in Africa, PMB, Kotoka International Airport, Accra, Ghana; 3 Faculty of Geo-Information Science and Earth Observation (ITC), Department of Urban and Regional Planning and Geo-Information Management, University of Twente, PO Box 217, 7500 AE Enschede, The Netherlands; 4 Laboratory of Geo-Information Science and Remote Sensing, Department of Environmental Sciences, Wageningen University and Research, P.O. Box 47, 6700AA Wageningen, The Netherlands

**Keywords:** Tree crops, cocoa, oil palm, mosaic landscape, intensity analysis, land-cover transitions, Ghana

## Abstract

Tree crops such as cocoa and oil palm are important to smallholders’ livelihoods and national economies of tropical producer countries. Governments seek to expand tree-crop acreages and improve yields. Existing literature has analyzed socioeconomic and environmental effects of tree-crop expansion, but its spatial effects on the landscape are yet to be explored. This study aims to assess the effects of tree-crop farming on the composition and the extent of land-cover transitions in a mixed cocoa/oil palm landscape in Ghana. Land-cover maps of 1986 and 2015 produced through ISODATA, and maximum likelihood classification were validated with field reference, Google Earth data, and key respondent interviews. Post-classification change detection was conducted and the transition matrix analyzed using intensity analysis. Cocoa and oil palm areas have increased in extent by 8.9% and 11.2%, respectively, mainly at the expense of food-crop land and forest. The intensity of forest loss to both tree crops is at a lower intensity than the loss of food-crop land. There were transitions between cocoa and oil palm, but the gains in oil palm outweigh those of cocoa. Cocoa and oil palm have increased in area and dominance. The main cover types converted to tree-crop areas are food-crop land and off-reserve forest. This is beginning to have serious implications for food security and livelihood options that depend on ecosystem services provided by the mosaic landscape. Tree-crop policies should take account of the geographical distribution of tree-commodity production at landscape level and its implications for food production and ecosystems services.

## Introduction

Landscapes face increasing pressure to produce more food and fiber commodities to meet the rising demands of an ever-growing global population (Meyfroidt et al. 2014; Laurance et al. 2014). The growing expansion of agricultural commodities occurs at the detriment of biodiversity, carbon storage, water preservation, and other ecosystem services needed to ensure food security and livelihoods (e.g., Hurni et al. 2015; Pradhan et al. 2015). A significant part of these commodities are tree crops such as oil palm, cocoa, and coffee, which target high-end markets in the global North (Ofori et al. 2014; Ros-Tonen et al. 2015). Agricultural policies in developing countries focus primarily on value chain integration in these high-end markets (e.g., Ros-Tonen et al. 2015).

In Ghana, for instance, the government aims to modernize tree-crop production and increase its productivity to benefit from growing global demand, and to boost employment and food security (Asante-Poku and Angelucci 2013; Angelucci 2013; MASDAR 2011; MoFA 2012). Cocoa and oil palm are leading crops in terms of area (1.63 Mha and 397,000 ha, respectively) and farmer participation (794,129 and 520,100 households, respectively) (GSS 2014). Cocoa is Ghana’s main export product, contributing 13.3% of the gross domestic product (GDP) (MoFA 2013). Oil palm is mainly absorbed by the local market as edible oil and crude oil in the soap industry, for which there are internal and regional market deficits of 32,000 tons and 450,000 tons, respectively (MASDAR 2011). Its contribution to Ghana’s GDP is negligible (1%) (Ibid.), but market deficits suggest growth potential. Increasing production areas and yields of these sectors are critical to achieving the objectives of Ghana’s tree-crop policy (MoFA 2012).

The consequences of tree-crop expansion for landscape components and multifunctionality are not well understood. This expansion implies encroachment into forests and conversion of other land-cover types (Lambin and Meyfroidt 2011). In particular, cocoa expansion is considered a key driver of deforestation in Ghana (Gockowski and Sonwa 2011; MLNR 2012) and literature suggests trade-offs between tree-crop production (oil palm and cocoa) and food security (Anderman et al. 2014). Earlier studies have assessed the effects of commodity-driven landscape change on livelihoods (e.g., Castella et al. 2013), but the main analysis focused on non-spatial aspects. Existing literature has also addressed landscape composition change in general (e.g., Margono et al. 2012; Antwi et al. 2014; Kusimi 2015), but studies that looked at the spatial-temporal dynamics of the expansion of agricultural commodities in mosaic landscapes are scarce (Abdullah and Nakagoshi 2006). The few studies addressing variations in tree-crop landscapes tend to amalgamate tree crops (cocoa and oil palm) with open forest (Alo and Pontius 2008; Adjei et al. 2014; Antwi et al. 2014; Kusimi 2015) or other forms of agriculture (Akinyemi 2013; Barima et al. 2016; Koranteng et al. 2016; Hackman et al. 2017). Furthermore, there is little knowledge about the degree of conversions among cocoa and oil palm, and to other land uses (losses) or vice versa (gains), and the respective spatial patterns and processes underlying transitions in such landscapes. Anecdotal evidence suggests however that in some regions cocoa is being converted to other land uses due to declining economic returns (Asante-Poku and Angelucci 2013).

This study aims to address these knowledge gaps by analyzing the landscape compositional dynamics in Ghana’s high forest zone due to the expansion (or shrinkage) of tree-crop cultivation. It specifically addresses the question of how tree-crop expansion drives the spatial transitions in the mosaic landscape in the Eastern Region of Ghana.

The remainder of the paper is structured as follows. The Materials and Methods section describes the landscape under study, the materials used (mainly two satellite images), and their properties. It further elaborates on the pre-processing, image classification, change detection, and the analysis of the transition matrix. The results section primarily presents the findings in terms of changes in landscape composition, land-cover change, and the intensities of change, further interpreted and compared with the broader literature in the discussion section. The study concludes with answering the main question and highlighting implications and suggestions for future research and landscape governance.

## Materials and Methods

### Study Area

**Fig. 1:**
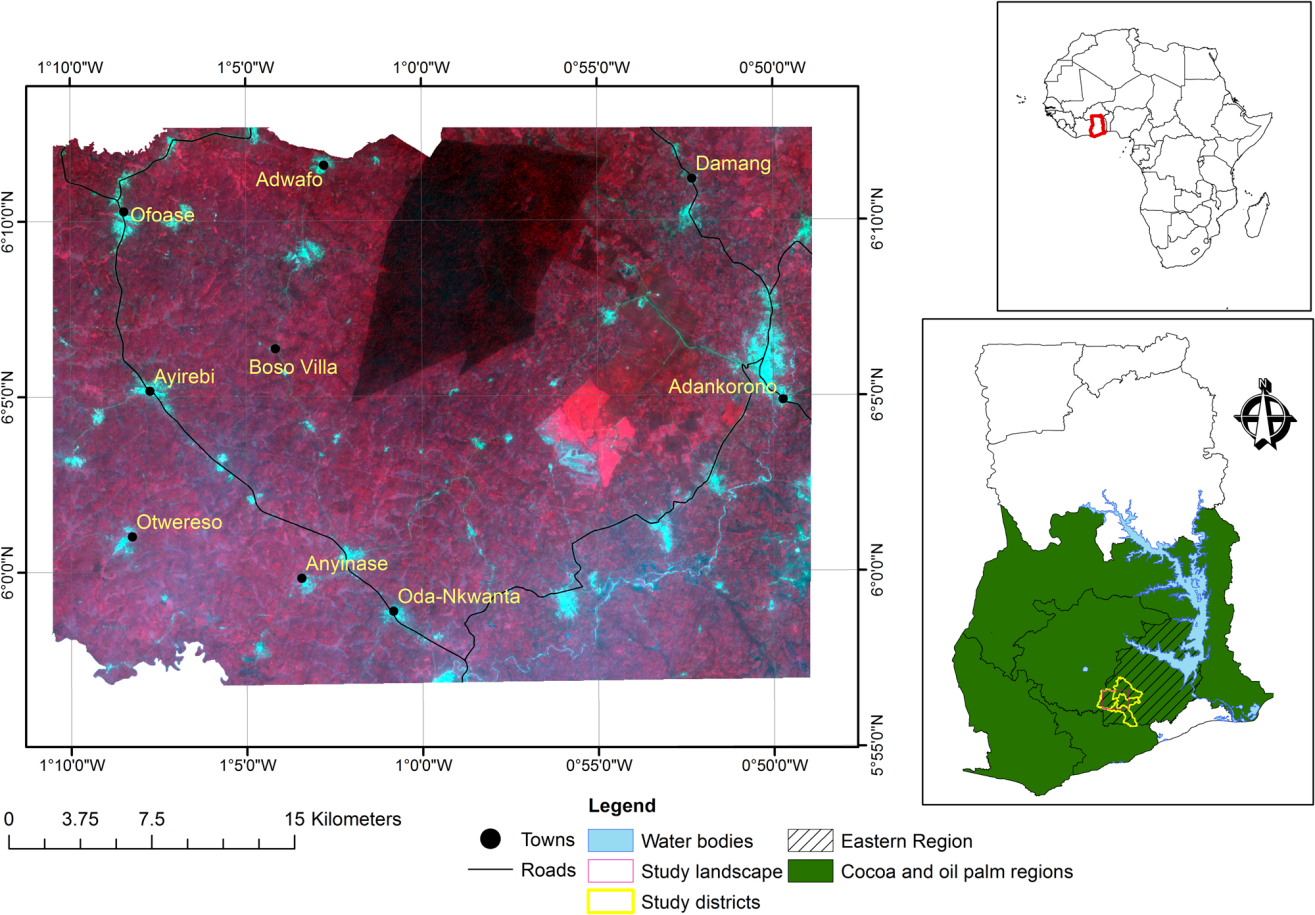
Location of the Akyemansa–Kwaebibrem landscape in the cocoa- and oil palm- growing regions in Ghana (Shapefile sources: Ghana at a glance, EPA and DIVA-GIS)

The study area is a mosaic landscape located in the southwestern corner of Ghana’s Eastern Region between latitude 5° 34’17”N and 6° 15’25”N, and longitude 0°47’10” W and 1° 9’55”W. It covers four adjoining administrative areas—Akyemansa, Denkyembour, and Kwaebibirem Districts—and Birim Central Municipality (Fig. 1). The landscape is hereafter referred to as the Akyemansa–Kwaebibrem landscape. Key towns and villages within this landscape include Kade, Ofoase, Ayirebi, Otwereso, Wenchi, Soaba, and Damang.

Ecologically, the area lies within the semi-deciduous forest ecological zone of Ghana and constitutes an integral part of the Guinea-Congolian biodiversity hotspot stretching along the West African coast (Hall and Swaine 1981). This ecological zone is affected by considerable transformation due to mining, agriculture, and settlement. The resulting landscape is a mosaic of tree crops, annual crops, remnants of the original closed canopy forest maintained as forest reserves, and patches of off-reserve forest.

The area experiences relatively large quantities of rainfall, a characteristic of the wet semi-equatorial climatic zone. Precipitation is bimodal, giving rise to two main raining seasons—March to July and August to October—with average rainfall ranging between 1500 mm and 2000 mm. Annual mean minimum and maximum temperatures are 25 °C and 27 °C, respectively. The landscape is noted for moderate to high relative humidity ranging between 55% in the dry season to 70% during the wet season (ERCC 2016). These climatic conditions have been fundamental to the success of tree-crop agriculture in the area (Michel-Dounias et al. 2015). Agriculture is the mainstay of people’s livelihoods, employing about 74% of the population (ERCC 2016).

The selected landscape presents favorable environmental conditions for the production of the focal tree crops, cocoa and oil palm. The area had some cocoa farms prior to 1940, while oil palm plantations developed since the late 1970s, and both have coevolved in the landscape till present (Michel-Dounias et al. 2015). This makes the area suitable for the study of the combined landscape effects of these crops.

### Data and Materials

Data used for the study includes two satellite images from Landsat (Table 1), land-cover reference data from fieldwork and Google Earth, and land-cover descriptions obtained through key informant interviews with chiefs and elderly people. The interviews were also analyzed for local perceptions of the change process, implications for ecosystem services, and future outlook of the Akyemansa–Kwaebibrem landscape. Landsat images were selected because of their geographic coverage of the study area, availability of a historical archive since the 1970s, and medium spatial and multispectral resolution, which all together makes them suitable for vegetation change detection at landscape scale (Xie et al. 2008; Vittek et al. 2014). Landsat images are also freely accessible from online data clearing houses. A random visual assessment of image tiles in this location revealed heavy presence of clouds and haze posing data utility constraints.

**Table 1:** List of Landsat images acquired for the study

Landsat scene ID	Acquisition date	Satellite/sensor	Path /row	Spatial resolution	No. of bands
LT51940561986363XXX09	1986/12/29	Landsat 5 / Thematic Mapper (TM)	194/056	30 m	7
LC81940562015363LGN00	2015/12/29	Landsat 8 / Operational Land Imager and the Thermal Infrared Sensor (OLI/TIRS)	194/056	30 m	11

A total of 158 surface reflectance images covering the period 1986–2015 were downloaded from the United States Geological Survey online databases through the Earth Explorer online data platform (http://earthexplorer.usgs.gov/). All the 158 images underwent pre-data selection processes using R 3.3.2 software to select those image datasets that fulfilled the following criteria: distribution and minimal area lost to clouds and thick haze, data quality, and being captured in the same season. A C-Language Function of Mask layer served to mask out clouds and then the Normalized Difference Vegetation Index was computed from all datasets to assess the quality of differentiating vegetation types. Two of the images, 1986 and 2015 respectively, emerged as being suitable for further analysis (Table 1). Both images were anniversary images captured in the dry season and this reduced anomalies in reflectance resulting between the datasets from phenological changes and sun angle differences (Fichera 2012; Wondrade et al. 2014). Preferably more than two time point images and wet season images are needed for trends analysis and differentiating various vegetation types (Wondrade et al. 2014; Diwediga et al. 2017), but the limited availability of cloud- and thick haze-free images restricted the image selection dates and periods to the dry season.

Historical data for both training and validating the classification of the 1986 Landsat 5 Thematic Mapper (TM) image was not available (Diwediga et al. 2017). In the absence of historical maps, knowledge about landscape composition in 1986 was obtained through key respondent interviews with 30 chiefs and elderly people from Ofoase, Ayirebi, Kade, and Soabe. To identify the geographical location of land-cover types, unchanged areas in 2015 were identified and applied to the classification of the 1986 image. Subsequently, image interpretation elements (tone, color, shape, and pattern) were also applied to identify the spread of land-cover types. A total of 49,454,863 pixels (59 polygons) were collected as reference data from “known” features within the unprocessed 1986 Landsat 5 TM image and from Google Earth images with a view to assessing quality of the classification.

**Fig. 2:**
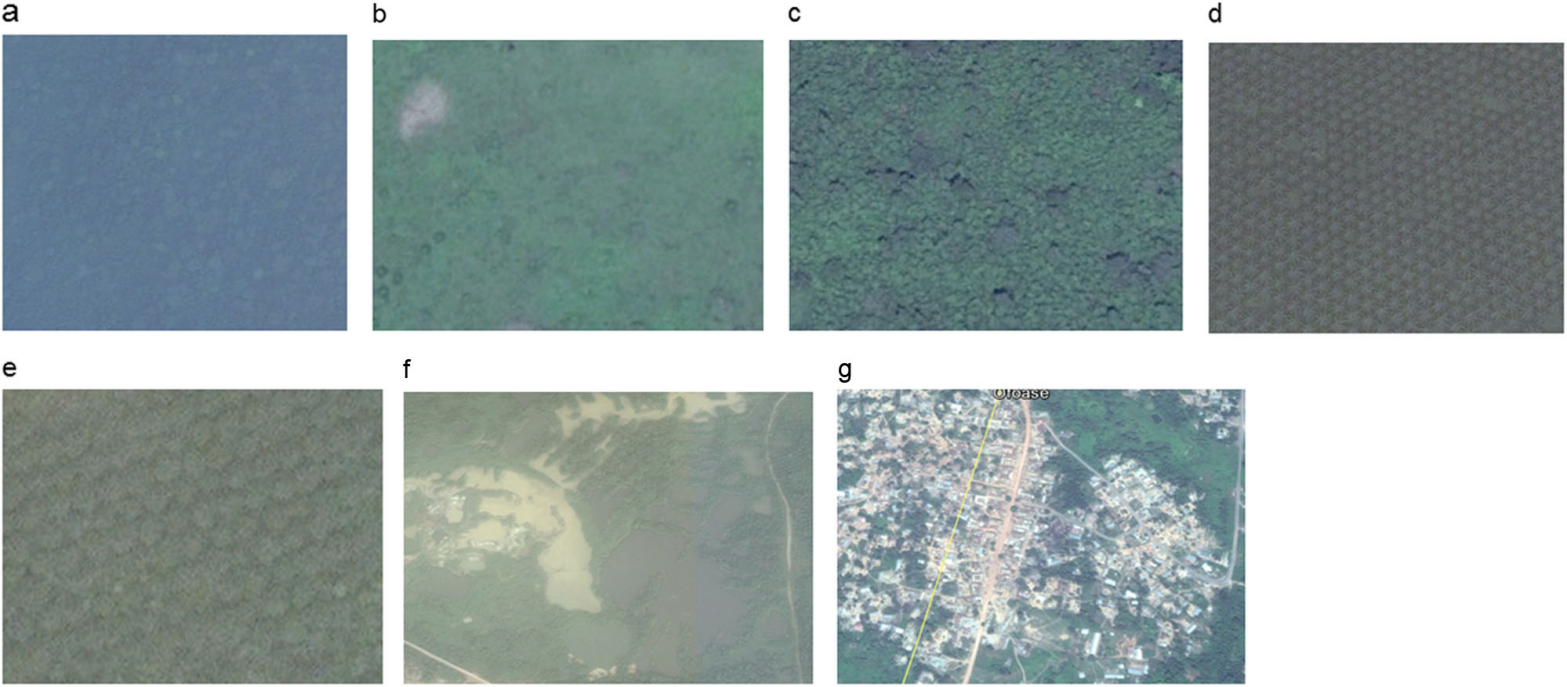
Clips of land-cover types (extracted from Google Earth), representing **a** forest, **b** food crop and fallow, **c** cocoa, **d** oil palm, **e** citrus, **f** water, and **g** built-up/bare area

Unlike the 1986 image, reference data (in total 908 polygons) for the 2015 Landsat 8 Operational Land Imager (OLI) image was gathered through field data collection and data extraction from Google Earth. Field data was collected by mapping representative polygon sample units of the identified land-cover types in the landscape with a handheld Global Positioning System. Attribute data included land-cover types, description of the surrounding environment, digital photos, and, where possible, oral information about changes. An area approach rather than a single point location was used to ensure that the sampled areas coincided with the respective land-cover types on the image. The field samples were augmented with 313 additional polygons extracted from high spatial resolution Google Earth images to cover areas that were inaccessible on the ground (Diwediga et al. 2017). The extraction was done through visual identification of recognizable representative land-cover types and on-screen manual digitizing in the Google Earth platform (Fig. 2). All the 908 polygons from both sources were combined, sorted by land-cover types, and systematically divided into training (469) and validation (439) datasets by selecting every other sample into either group.

### Land-cover Mapping

**Fig. 3:**
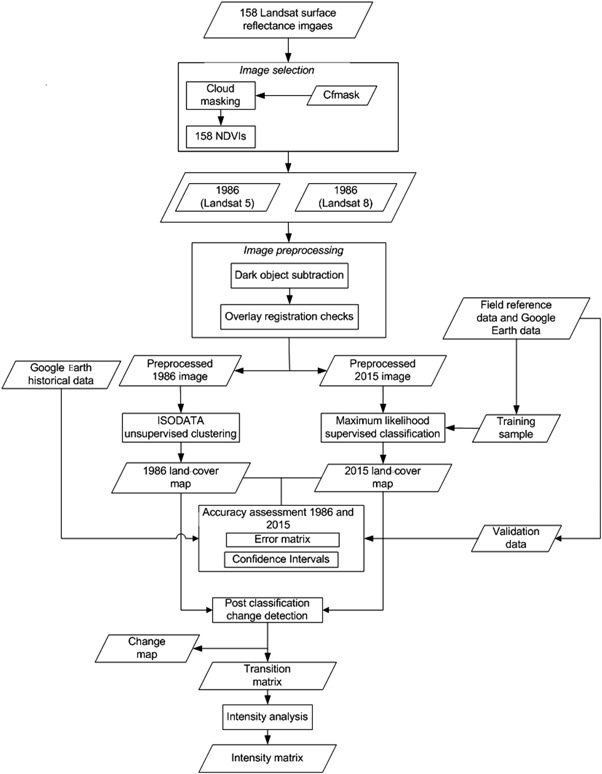
The land-cover mapping process

The process of land-cover mapping includes development of the land-cover classification scheme, image pre-processing, and image classification (Fig. 3).

There is no unique land-cover classification scheme for Ghana. Available schemes differ according to the research objective of the various landscape studies. This study developed a land-cover classification scheme drawing on Asubonteng (2007), Adjei et al. (2014), and Asare et al. (2014), while consciously including context-specific features in the study area (cocoa, oil palm, and food crops). The scheme is both locally relevant and fits the research focus on tree-crop farming (Table 2).

**Table 2:** Land-cover classification scheme used for the study

Land-cover type	Description
Built-up/bare	Areas with high and low intensities of infrastructural development and exposed soil surfaces with little or no capacity to support plant life. This class includes roads (tarred and untarred), towns, waste lands, and rock outcrops.
Food crops	Land primarily used for the production of food, mainly annual and biannual crops. It also includes natural vegetation areas that oscillate between production and fallow periods in a food production cycle. The latter are predominantly grass and shrubs.
Cocoa	Small- to large-scale cocoa farms of different tree densities and age categories
Oil palm	Small- to large-scale oil palm farms of different tree intensities and age categories. Naturally occurring oil palm along water bodies is included in this class.
Other tree crops	Comprises all other tree-crop plantations in the landscape, mainly rubber and citrus.
Forest	Naturally growing woody tree vegetation clusters with stems reaching 5 m height. Bamboo clusters and timber plantations are included.
Water	All forms of exposed water surfaces including rivers, reservoirs, and ponds.

Pre-processing refers to initial operations administered to the raw satellite images to remove or reduce errors due to sensor and platform-specific radiometric and geometric distortions. Atmospheric defects are common defects usually associated with most optical images of the tropics, including the southern highland areas of Ghana. However, the use of surface reflectance images pre-processed with Landsat Ecosystem Disturbance Adaptive Processing System and Landsat Surface Reflectance Code reduced geometric and atmospheric effects on Landsat TM and Landsat OLI, respectively. In addition, dark object subtraction was employed to reduce the effects of remaining haze on both images. In the absence of clearly visible water bodies for dark pixel reference value, minimum pixel values were subtracted from the images at the band level using ENVI 5.0 Classic software. Both the 1986 and 2015 images were spatially overlaid and swiped to ensure that they are co-registered and devoid of any spatial mismatch. Spatial overlay is essential in a change-detection process to ensure that changes detected represent actual changes in pixel classes between the compared images and are not due to registration error between the images. Finally, both images were subsetted to the extent of the landscape.

Land-cover classification encompasses the process of grouping similar pixels into a class and assigning them land-cover type labels (Campbell and Wynne 2011). Different classification approaches were applied to 1986 and 2015 due to the difference in data availability for both images. Iterative Self Organizing Data Analysis (ISODATA) unsupervised classification was used for the 1986 image due to the unavailability of an original historical map and field reference data, whereas supervised maximum likelihood classification (MLC) was used in the case of the 2015 image because of the readily available field reference data.

Using ISODATA unsupervised classification algorithm in ENVI 5.0 classic software, the 1986 image was classified into 80 initial classes using the classification scheme presented in Table 2, which allows the natural grouping of pixel values in images. The larger number of classes beyond the desired number made it possible to separate closely related, but different classes within the landscape. The classes were further aggregated into the seven classes listed in Table 2. Identification of land-cover types and clustering into final classes were based on unchanged land-cover types between 1986 and 2015 (Wondrade et al. 2014; Diwediga et al. 2017), and the use of image interpretation elements (Campbell and Wynne 2011).

Spectral signatures were extracted from the 2015 image using the training dataset (469) and applied in a MLC in ENVI 5.0 Classic software to categorize the image in predefined land-cover types in the landscape. Known sites of mixed young oil palm that were misclassified as forest in the 2015 image were manually digitized on screen into their correct classes (oil palm) to improve the accuracy and utility of the map (Zhou et al. 2014).

Post-classification accuracy assessment was performed as an integral part of the classification processes of both images. Validation datasets for the 1986 and 2015 images were used as reference to assess the quality of the thematic land-cover maps of the respective years. The resultant pixel-based error matrices from the accuracy assessment were converted to area proportions of the landscape to recalculate a new set of accuracy parameters considering the stratification (Olofsson et al. 2014). Uncertainties and adjusted areas were also computed for both land-cover maps.

### Change Detection

Change detection was carried out to identify and assess transformations that have occurred in the landscape over the 29-year period under study. The assessment focused on proportions of change and rates, spatial distribution of change, transitions over time (Lu et al. 2004), and changes in the land-cover composition of the landscape. Post-classification change detection technique was employed to compare the independently produced land-cover maps for 1986 and 2015 (Dalle et al. 2011; Fichera 2012; Zhou et al. 2014). Although being time-consuming and sensitive to the combined errors from both images, the technique is the most commonly used in land-cover change monitoring and assessment literature (e.g., Fichera 2012; Adjei et al. 2014; Wondrade et al. 2014). The technique’s ability to reduce effects of sensor, atmospheric, and environmental differences on the outputs and the generation of a transition matrix made it a suitable choice for the study (Coppin et al. 2002; Lu et al. 2004; Tewkesbury et al. 2015). The image pair resulted in a land-cover change map and a transition matrix. The matrix consists of the sizes of land-cover units in the initial year and the conversions that have occurred until the end year. The values in the diagonal of the transition matrix represent persistent land-cover areas per category, whereas the off-diagonal values are the transitions in land-cover types during the 29-year period. The matrix includes gross losses, gross gains, net changes and swaps to generate further insights into the nature of changes in the landscape (Pontius et al. 2004; Alo and Pontius 2008). Gross losses refer to the sum of all proportions per individual land-cover type in 1986 that had changed to other land-cover types by 2015 and is calculated by subtracting the persistence from the total in the right-hand column (Pontius et al. 2004). Gross gains are the sum of all areas per land-cover type converted from other land-cover types by 2015; the column totals minus the persistence (ibid.). The absolute difference between gross gain and gross loss is the net change. A swap occurs when a land-cover type gains in one location and loses equal amounts in one or more other locations in the landscape. A swap is calculated as the difference between the total change and the net change between 1986 and 2015 (Pontius et al. 2004; Alo and Pontius 2008).

The annual rate of change (R) was computed for all land-cover components in the landscape using the widely adopted synergized formula drawn from the compound interest law and mean annual rate of change R (Puyravaud 2003).

1 $$R = \left( {\frac{1}{{\left( {t_2 - t_1} \right)}}} \right)\,\ln \left( {\frac{{A_2}}{{A_1}}} \right)$$

Where *A*_1_ and *A*_2_ are the areas of land-cover types at time *t*_1_ and *t*_2_, respectively. A negative signed rate of change represents a decline in area per year, whereas positive rates are an increase in area per year over the researched period.

### Intensity Analysis

The transition matrix of the change detection provides a general overview of land-cover stocks in the landscape and transfers among land-cover categories between 1986 and 2015. However, it fails to reveal the underlying processes that drove transition patterns in the landscape between 1986 and 2015 (Pontius et al. 2004), which may lead to erroneous policy recommendations. Intensity analysis was applied to generate insights into the patterns and processes in the observed changes among tree crops and other components in the mosaic landscape (Aldwaik and Pontius 2012). This quantitative framework explains the dynamic land-cover transitions in the same location between different time points at three levels: interval, category, and transition (Aldwaik and Pontius 2012). Analysis at interval level considers differences in land-cover change between different time intervals—not applicable here because we consider one time interval only. The intensity analysis in this study was implemented at the category and transition levels, respectively, using spreadsheets based on Aldwaik and Pontius’s (2012) equations. The analysis at category level focuses on the size and intensity of gross gains and losses per land-cover category over the time period under study. Transition level analysis assesses the variation in size and intensity of specific transitions among the categories that are available for that transition. Both category and transition level intensities are compared with uniform intensity lines, which represent the value under a hypothetical condition of uniform distribution of change across all categories. If a category intensity exceeds that of the uniform line, then the change of that category is deemed relatively fast (referred to as active). If it does not reach the uniform line, the transition is relatively slow (referred to as dormant) during the given time interval (Zhou et al. 2014). Similarly, if a particular gaining or losing category has a transition intensity that passes the uniform intensity line it is considered to be targeting specific land-cover categories, and when it does not reach the uniform intensity line it is considered to avoid these categories (Aldwaik and Pontius 2012).

## Results

### Land-cover Mapping in 1986 and 2015

**Fig. 4:**
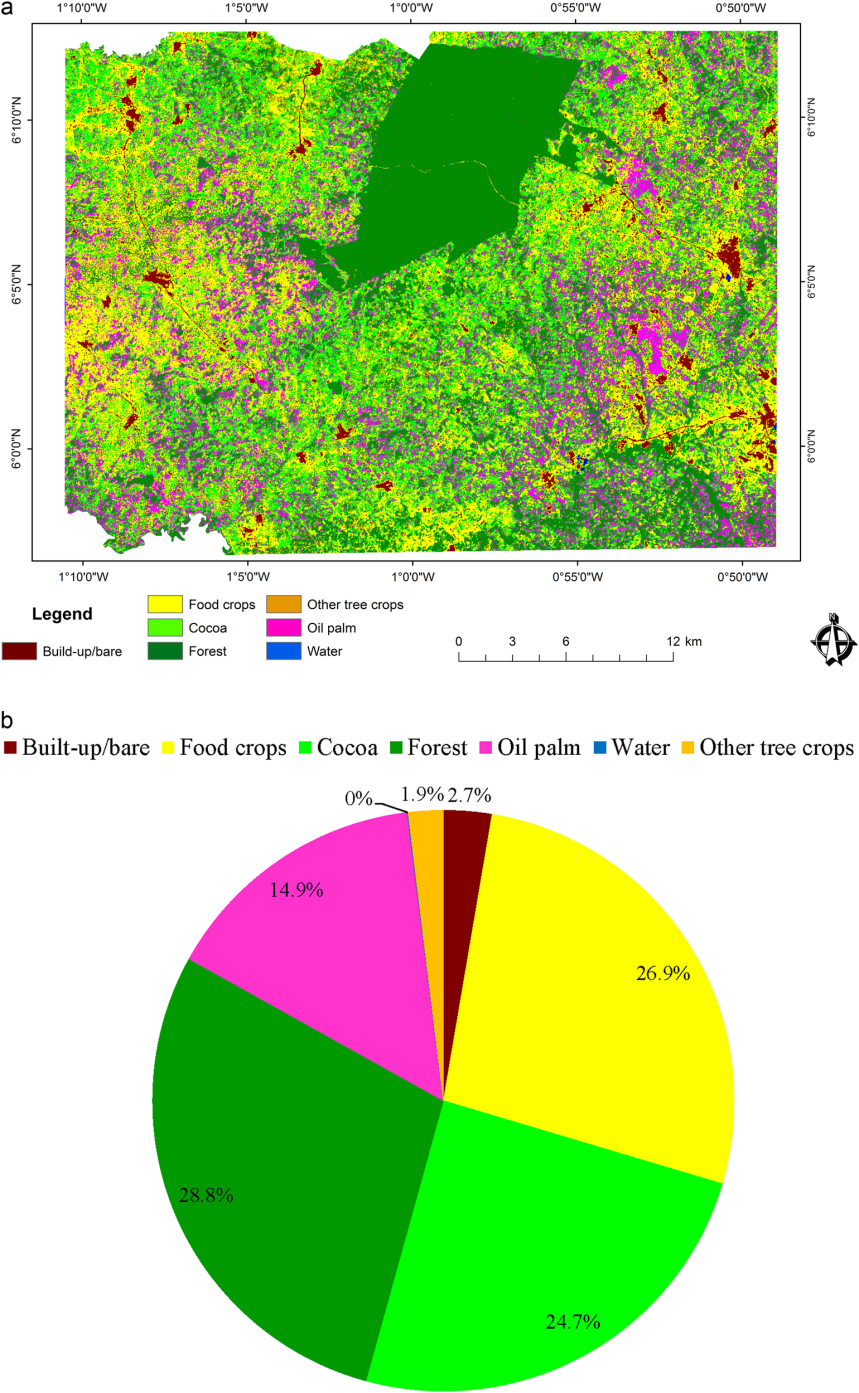
**a** Classified land-cover map of 1986 (data source for classification: Landsat 5 1986). **b** Proportions of the land-cover types in the landscape

In 1986, the Akyemansa–Kwaebibrem landscape consisted of seven main land-cover types of varying proportions and spatial distribution (Fig. 4), with forest (28.8%), food crops (27%), and cocoa (24.7%) dominating, and oil palm occupying a mere 14.9%. Two main forest areas were located in the north and southwestern corner of the landscape, respectively, with the first being the largest patch of contiguous forest in the landscape. The central portion was made up of cocoa interspersed with forest and a few food-crop patches, whereas larger areas of food crops occurred in the west and oil palm clusters were found scattered over the eastern part of the studied landscape. Food-crop land surrounded human settlements and transportation networks constituting built-up/bare areas were spreading outwardly at varying extents. Exposed water surface was insignificant in the landscape.

**Fig. 5:**
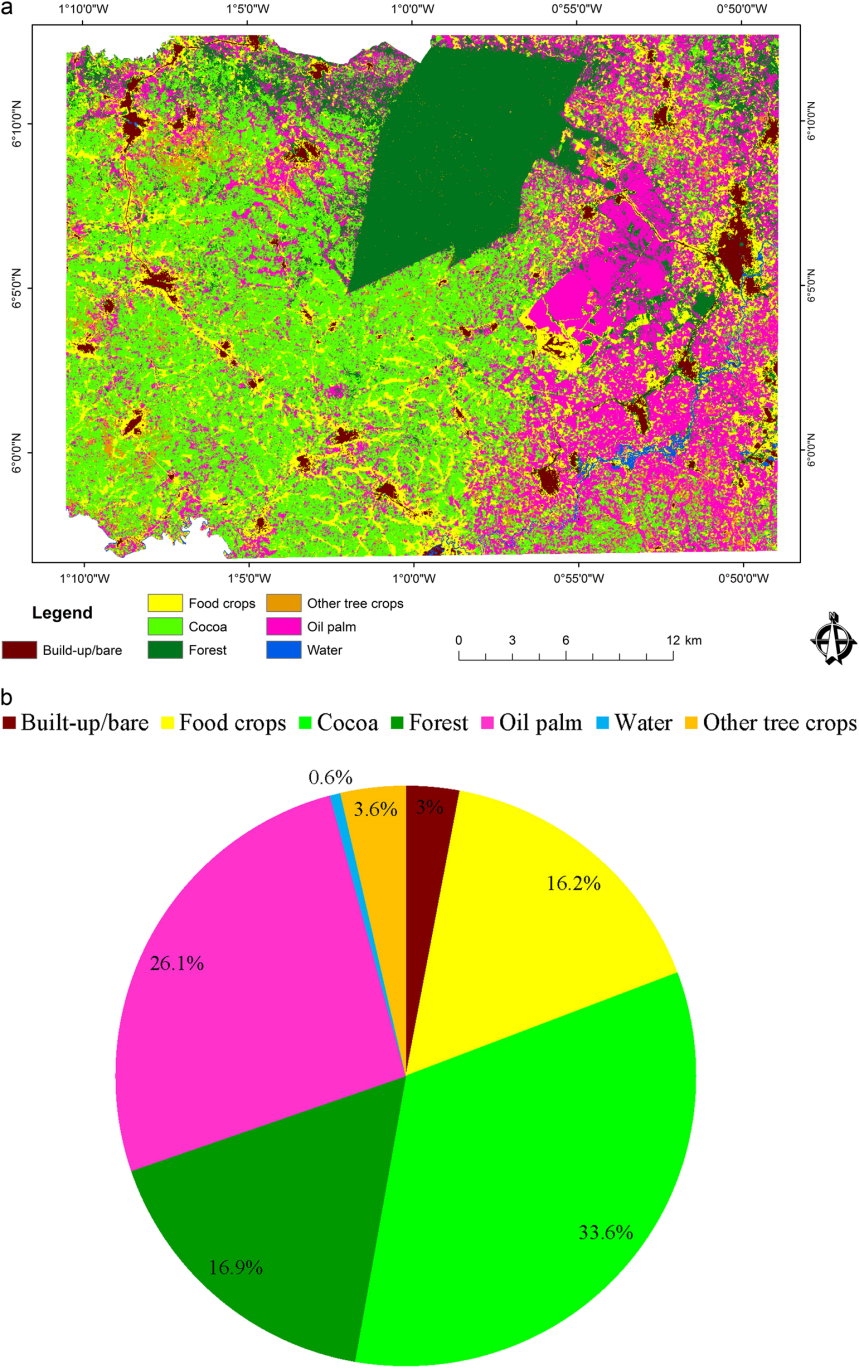
**a** Classified land-cover map 2015 (data source for classification: Landsat 8 2015). **b** Proportions of the land-cover types in the landscape

In 2015, the key components of the landscapes had remained the same, but changes had occurred in both the spatial distribution and areal extents of the land-cover types (Fig. 5), with cocoa (33.6%) and oil palm (26.1%) having become the prevailing land-cover types in 2015, followed by forest (16.9%) and food crops (16.2%). The minor land-cover types were other tree crops (3.6%), built-up/bare (3%), and water (0.6%). In 2015, the landscape was divided into three main areas with cocoa stretching from the west to middle, oil palm being mainly in the east, and forest concentrated in the central-north of the landscape. Beyond these broader divisions, a few patches of forest, cocoa, and oil palm were scattered across the landscape devoid of any unique pattern. Food crops and trees other than cocoa and oil palm are found in smaller patches interspersing with cocoa and oil palm areas. Water runs through the oil palm areas on the eastern side with some ponds on the river banks. Built-up/bare areas have a line-and-node pattern running diagonally from the north-western corner to the middle central part of the landscape, and another one running to the north eastern corner (Figs. 4a and 5a).

The pixel-based overall accuracies of the 1986 and 2015 land-cover maps were 91.2% and 78.8%, respectively (Table 3). However, the estimated area proportions reduced the accuracies of both years by about 6%. The accuracy of the 1986 classified map aligned better with the respective reference data than the 2015 classified map. For water, the study recorded the lowest producer accuracy (4%) in the 1986 map, whereas in 2015 other tree crops had low producer (45%) and user (49%) accuracies. Uncertainties in land-cover type areas in both maps and their adjusted areas are found in Table 7 in the Appendix.

**Table 3:** Summary of accuracy estimates based on area proportion estimates for 1986 and 2015

	1986 Land-cover map						
	Built-up/bare	Food crops	Cocoa	Oil palm	Other tree crops	Forest	Water
Overall accuracy = 85.2%; *91.2							
User^a^	96%	78%	82%	80%	91%	95%	100%
Producer^b^	94%	90%	92%	83%	53%	83%	4%
	2015 Land-cover map						
	Built-up/bare	Food crops	Cocoa	Oil palm	Other tree crops	Forest	Water
Overall accuracy = 73.0%; *78.8							
User	97%	72%	73%	89%	49%	50%	85%
Producer	95%	68%	91%	64%	45%	68%	92%

^a^User accuracy refers to errors of commission (e.g., erroneously including cocoa in the forest category)

^b^Producer accuracy refers to errors of omission (e.g., excluding water hidden under canopy)

### Land-cover Change, Change Rates, and Landscape Transitions

Table 4 displays stock changes between 1986 and 2015 and their rates of change. Comparing surface areas between 1986 and 2015 shows that all land-cover types have undergone varying degrees of change in size at different rates. Forest and food-crop land coverage declined by 11.9% (135.14 km^2^) and 10.7% (121.5 km^2^), respectively. With an annual change rate of 1.84%, forest accounts for the largest change in the landscape. Food-crop land changed at an annual rate of 1.75% in a decreasing direction. All other land-cover types increased in size and proportion. Oil palm recorded the largest positive change in land-cover size (11.2%), followed by cocoa (8.9%), other tree crops (1.67%), water (0.55%), and built-up area (0.3%). Changes in oil palm and cocoa occurred at 1.93% and 1.06% per annum, respectively. Considering their initial extent, water (0.44 km^2^) and other tree crops (22.04 km^2^) changed at rates of 9.4% and 2.1%, respectively to achieve their sizes in 2015 (Table 4). Water recorded the fastest rate in the landscape. The smallest change rate was seen in the built-up area (0.4%).

**Table 4:** Stock changes and annual change rates of land-cover types

Land-cover type	1986 area (km^2^)	2015 area (km^2^)	Change (km^2^)	Change (% of landscape area)	Annual change rate (%)
Built-up/bare	30.35	33.75	3.4	0.30	0.37
Food crops	305.75	184.25	− 121.5	− 10.71	− 1.75
Cocoa	280.01	381.26	101.25	8.92	1.06
Oil palm	169.19	295.99	126.8	11.18	1.93
Other tree crops	22.04	40.99	18.95	1.67	2.14
Forest	326.74	191.6	− 135.14	− 11.91	− 1.84
Water	0.44	6.68	6.24	0.55	9.38

The landscape transition matrix (Table 5) demonstrates the gross gains and gross losses, outlining the details of land transfers among land-cover types over the 29 years period. The persistence in the landscape amounts to 377.8 km^2^ constituting 33.3% of the entire landscape, implying that 66.7% of the landscape was subject to land transitions. Gross gains in a land-cover type means an equal area of gross loss in other land-cover types. The landscape has a relatively high change-to-persistence ratio, a sign of high dynamism in the landscape. Major transitions within the landscape are food crops–cocoa–food crops, food crops–oil palm–food crops, forest–cocoa–forest, and cocoa–oil palm–cocoa.

**Table 5:** Land transitions in km^2^ (top values) and in percentage of the total landscape (bottom values in bold)

Land-cover types	Built-up/bare	Food crops	Cocoa	Oil palm	Other tree crops	Forest	Water	1986 Total
Built-up/bare	13.78	6.93	2.01	4.66	0.48	1.98	0.51	30.35
	**1.21**	**0.61**	**0.18**	**0.41**	**0.04**	**0.17**	**0.04**	**2.68**
Food crops	12.43	70.61	103.92	81.68	12.73	23.29	1.09	305.75
	**1.10**	**6.22**	**9.16**	**7.20**	**1.12**	**2.05**	**0.10**	**26.95**
Cocoa	4.16	52.03	108.85	79.63	11.30	23.65	0.39	280.01
	**0.37**	**4.59**	**9.59**	**7.02**	**1.00**	**2.08**	**0.03**	**24.68**
Oil palm	1.15	21.45	69.91	56.78	7.23	12.28	0.39	169.19
	**0.10**	**1.89**	**6.16**	**5.00**	**0.64**	**1.08**	**0.03**	**14.91**
Other tree crops	0.25	2.69	9.81	5.38	0.55	3.33	0.03	22.04
	**0.02**	**0.24**	**0.86**	**0.47**	**0.05**	**0.29**	**0.00**	**1.94**
Forest	1.98	30.42	86.76	67.83	8.60	127.03	4.12	326.74
	**0.17**	**2.68**	**7.65**	**5.98**	**0.76**	**11.20**	**0.36**	**28.80**
Water	0.00	0.12	0.00	0.03	0.10	0.04	0.15	0.44
	**0.00**	**0.01**	**0.00**	**0.00**	**0.01**	**0.00**	**0.01**	**0.04**
2015 Total	33.75	184.25	381.26	295.99	40.99	191.60	6.68	1134.52
	**2.97**	**16.24**	**33.61**	**26.09**	**3.61**	**16.89**	**0.59**	**100.00**

The diagonal (underlined) indicates the persistence of land-cover types

By 2015, food-crop land lost 235.14 km^2^ (20.73%), accounting for the highest gross loss in the study area. It gained 113.64 km^2^ (10%) as the third highest gross gainer in the landscape (Table 5), leaving a net negative change of 121.5 km^2^ (10.71%). In addition to the gains and losses, swaps amounted to 227.28 km^2^ (20.03%) (Table 6). The high disparity between swaps and net changes indicates large location exchanges of food-crop land with other land-cover types. Significant replacements of food-crop land were mainly from cocoa (9.16%), oil palm (7.20%), and forest (2.05%). In spite of the swaps and net changes in food-crop land, 6.22% of the landscape was maintained under persistent food-crop land between 1986 and 2015.

**Table 6:** Persistence, gain, loss, net change, and swaps (top values: area in km^2^; bottom values (in bold): % of the total landscape)

Land-cover type	Persistence (km^2^)	Gain (km^2^)	Loss (km^2^)	Gain + Loss	Net change (absolute)	Swaps
Built-up/bare	13.78	19.97	16.57	36.54	3.4	33.14
	**1.21**	**1.76**	**1.46**	**3.22**	**0.30**	**2.92**
Food crops	70.61	113.64	235.14	348.78	(−)121.5	227.28
	**6.22**	**10.02**	**20.73**	**30.74**	(−)**10.71**	**20.03**
Cocoa	108.85	272.41	171.16	443.57	101.25	342.32
	**9.59**	**24.01**	**15.09**	**39.10**	**8.92**	**30.17**
Oil palm	56.78	239.21	112.41	351.62	126.8	224.82
	**5.00**	**21.08**	**9.91**	**30.99**	**11.18**	**19.82**
Other tree crops	0.55	40.44	21.49	61.93	18.95	42.98
	**0.05**	**3.56**	**1.89**	**5.46**	**1.67**	**3.79**
Forest	127.03	64.57	199.71	264.28	(−)135.14	129.14
	**11.20**	**5.69**	**17.60**	**23.29**	(−)**11.91**	**11.38**
Water	0.15	6.53	0.29	6.82	6.24	0.58
	**0.01**	**0.58**	**0.03**	**0.60**	**0.55**	**0.05**

Likewise, forest recorded a loss of 199.71 km^2^ (17.6%). Conversions to forest land (5.69%) were less than to cocoa (24.01%), oil palm (21.08%), and food crops (10.02%), but significantly higher than conversions to built-up area (1,76%), other tree crops (3.56%) and water (0.58%). With a net change of 135.14 km^2^, forest was the land-cover category with the largest extent converted to other land-cover types. Swaps between forest and other land-cover types amounted to 129.14 km^2^. The relatively small difference between net change (11.91%) (deforestation) and swaps (11.38%) in forest indicates that deforestation in one place is compensated by reforestation in another place at a degree close to permanent tree-cover loss. These values are also close to the amount of forest that persisted in the landscape (11.20%), the largest value of all land-cover types (Table 6).

Contrary to food-crop land, cocoa recorded a gain of 272.41 km^2^ (24.01%) (Table 6). However, cocoa also lost considerable amounts (171.16 km^2^) to other land-cover types, as did food-crop land (235.14 km^2^) and forest (199.71 km^2^). High gains and losses make cocoa the most dynamic land cover. Of the change, swaps contribute 30.17%, whereas net gains amount to 8.92% of the landscape. Swaps in cocoa are the largest of all land-cover types, indicating shifts in location across the landscape. Gains in cocoa are coming from food-crop land (9.16%), forest (**7**.65%), and oil palm (6.16%), respectively, whereas losses in cocoa are due to conversion to oil palm (7.02%), food-crop land (4.59%), and forest (2.08%) (Table 5). This indicates a slight loss of land for cocoa to land for oil palm, with a net gain at the cost of forest and food-crop land. At the same time, second to forest, cocoa (9.59%) has a relatively high persistence in the landscape.

Similar to cocoa and conversely to forest and food-crop land, oil palm expanded considerably. It saw a net change of 11.18% (126.8 km^2^) of the total landscape and is therefore the land cover with the largest positive net change. This is a result of oil palm gaining more (21.08%; second largest) than losing (9.91%) during the transition period under study. Of the total change in oil palm, swapping accounts for more than half, equaling 19.82% of the landscape. Similar to cocoa, the largest contributions to oil palm came from food-crop land (7.20%), closely followed by cocoa (7.02) and forest (5.98) (Table 5). Contributions from built-up area (0.41%) and other tree crops (0.47) were marginal, whereas the water area experienced no conversion to oil palm.

### Intensity Analysis

**Fig. 6:**
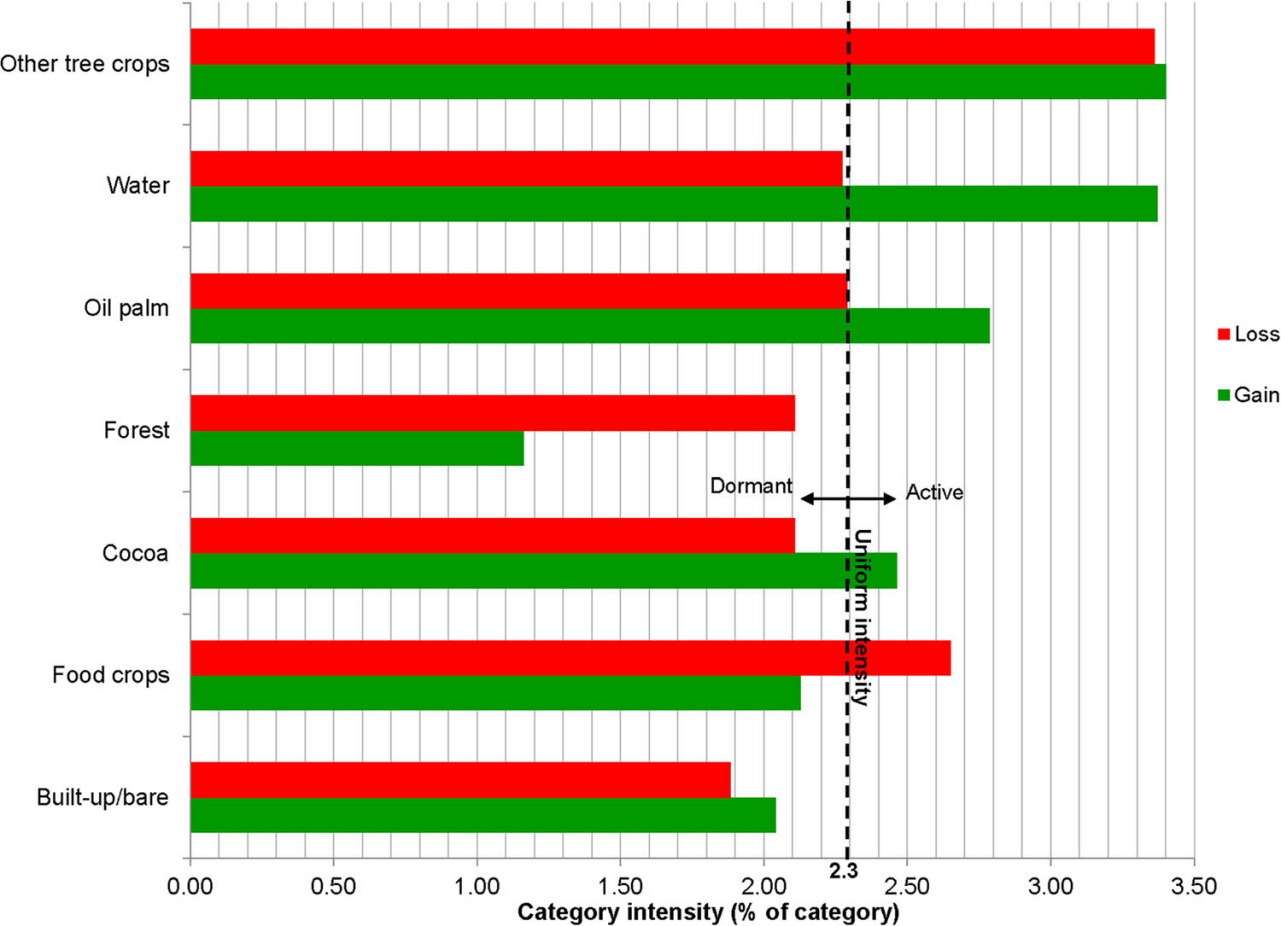
Category level intensities for 1986–2015

Category level intensity analysis determined the categories that experienced dormant or active changes when compared with the overall intensity of landscape change. Loss and gain intensities per category are graphically shown in Fig. 6.

Gains and losses for forest and built-up area were both dormant, whereas land for other tree crops (e.g., citrus) recorded intensively active gains and losses. Land with food crops experienced intense active losses and minimally dormant gains. Contrastingly, water, oil palm, and cocoa intensively gained, whereas their losses were dormant. Intensities of gains (active) and losses (dormant) are relatively higher for oil palm than for cocoa.

**Fig. 7:**
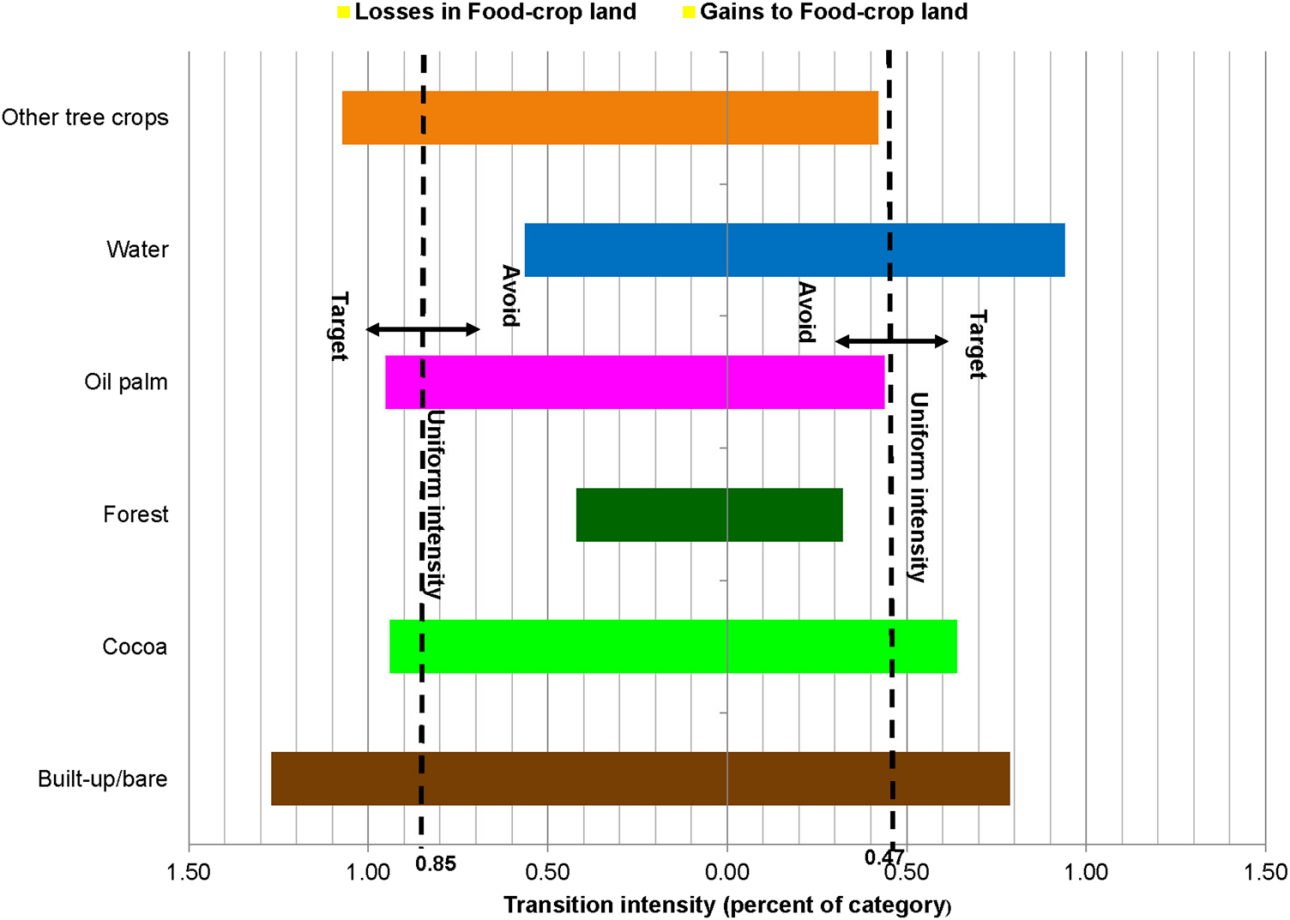
Transition level intensities for crop land (losses on the left and gains on the right)

**Fig. 8:**
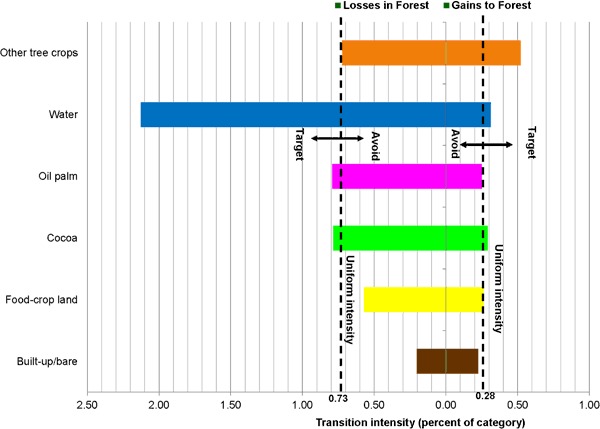
Transition level intensities for forest (losses on the left and gains on the right)

**Fig. 9:**
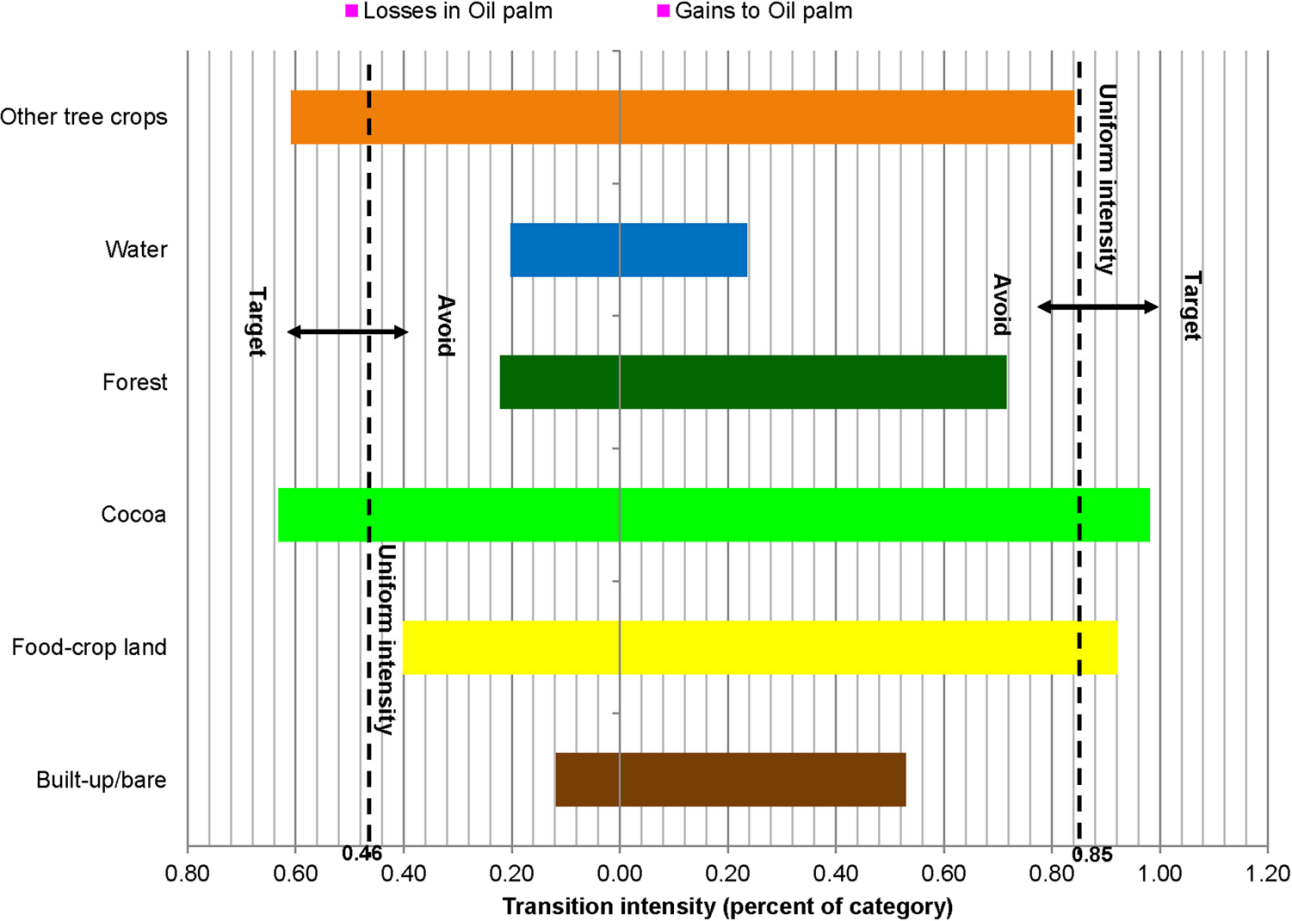
Transition level intensities for oil palm (losses on the left and gains on the right)

**Fig. 10:**
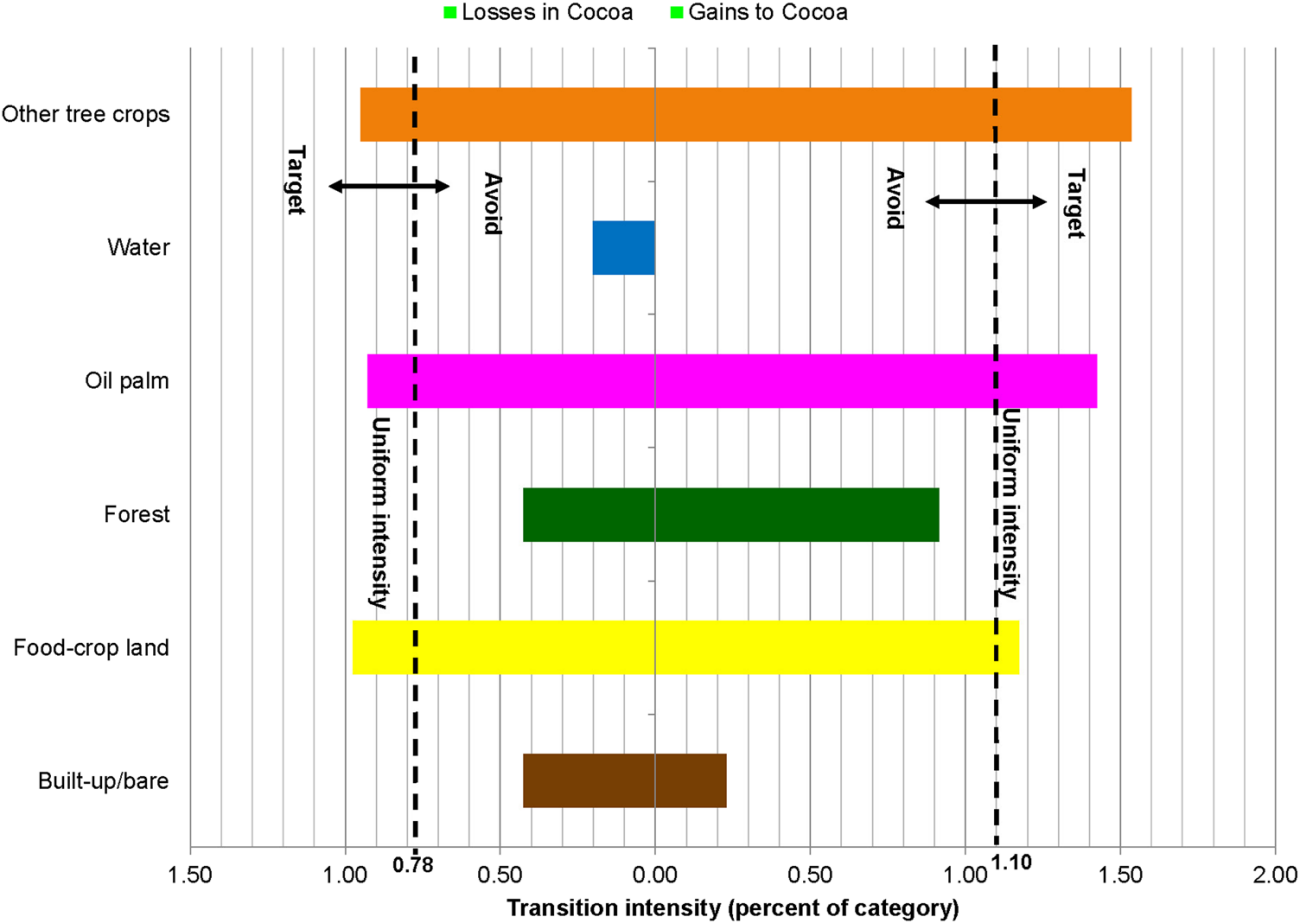
Transition level intensities for cocoa (losses on the left and gains on the right)

The paired bar charts in Figs. 7, 8, 9 and 10 compare observed transition intensities between land-cover types with hypothetical uniform intensities marked as vertical dash lines on either side of the charts. The left uniform intensity line represents the hypothetical uniform value for the intensity of transitions resulting in losses in the respective land categories; the right one is the uniform value for the intensity of transitions leading to gains in land categories.

Losses in food-crop land target all other land-cover types, except forest and water (Fig. 7), with transitions to built-up/bare areas being more intensive than those to tree crops (cocoa, oil palm, and other). Expansion of food-crop land avoids oil palm, other tree crops, and forest in decreasing order of intensities. Rather, it targets water, built-up/bare area, and cocoa to gain.

Forest loss targets oil palm and cocoa at similar intensities and avoids food-crop land, built-up/bare area and other tree crops (Fig. 8). Intensity was highest for the transition from forest to water. Forest gains target other tree crops and to a lesser extent water and cocoa at comparable intensities. Oil palm, food-crop land and built-up/bare area are marginally avoided.

Losses in oil palm area target cocoa and other tree crops at similar transition intensities, and avoid food-crop land, forest, water, and built-up/bare area in declining order (Fig. 9). Expansion of oil palm in the landscape targets cocoa and, at a lesser intensity, food-crop land, and avoids other tree crops slightly. Oil palm expansion avoids forest, built-up/bare area, and water, with transformation intensities in declining order.

Cocoa targets food-crop land, oil palm, and other tree crops for both losses and gains, but transition intensities gained from oil palm and other tree crops were higher than their respective losses. Forest, built-up/bare area, and water were avoided (Fig. 10). Water recoded zero transition for the expansion of cocoa.

### Landscape Dwellers’ Impressions

Cocoa farms are established through gradual reduction of tree shade by converting forests. In the case of food-crop land conversion, cocoa is nurtured under shade crops such as plantain. Upon maturity, food crops are excluded from the plots, except a native yam species, locally referred to as “kokooase bayere” (*Dioscorea* spp.). In wetter areas, oil palm is also established through conversion of riverine forest or rice fields. Although cocoa can exist on the same land for 25–50 years, oil palm farms usually are to last for about 25 years. Oil palm and cocoa lock up lands during these years and reduce lands available for food-crop production. Farmers cultivating these tree crops hardly switch to other crops. However, in situations where cocoa is failing in wetlands, it is replaced with rice or vegetables and eventually oil palm. The dominance of cocoa in the western part of the study area and of oil palm in the eastern part is determined by site suitability factors such as elevation, soil fertility, and water content, with lower elevations and wetter conditions being more favorable for oil palm.

Economic triggers will also determine the future composition of the landscape. For example, the relative prices of cocoa (set by the government), oil palm (to be negotiated with private companies), and food crops (generally low) influence farmers’ decisions about crop choices. Informants expect that cocoa and oil palm will continue to dominate the landscape and, together (but to a lesser extent) with built-up areas, will expand at the expense of other land-cover categories. Interviewees associate the loss of food-crop land with the expansion of the two major tree crops and the influx of migrant workers, and expansion of settlement areas in the vicinity of these lands. Farmers have resorted to growing food crops at distant locations away from rapidly growing settlement areas. They expect that the situation will lead to increased food shortages and food prices that are already experienced in the area, in particular during the lean season. Farmers are practicing backyard cropping and intercropping in the initial stages of tree-crop farming to supplement food purchased from distant markets. Considering these trends, they expect that the future forest in the landscape will be restricted to forest reserves and sacred areas.

## Discussion

### Land-cover Mapping and Accuracy

The study successfully applied conventional unsupervised (ISODATA) and supervised (MLC) algorithms to map food-crop land, forest, cocoa, oil palm, and other land-cover types in the mosaic landscape derived from 1986 and 2015 Landsat images, respectively. Contrary to earlier studies (Alo and Pontius 2008; Asare et al. 2014; Kusimi 2015; Hackman et al. 2017), this study separated cocoa from forest and other vegetation types in the landscape. It further enabled a finer scale of land-cover mapping in the landscape and analysis of the land transfers among them using conventional classifiers, which was a gap in existing studies.

The estimated overall accuracies (area proportions) of 1986 and 2015 land-cover maps produced from ISODATA and MLC classifiers respectively were relatively lower than those derived from the pixel count error matrix, but comparable with other studies that used coarse categories in similar landscapes (e.g., Kusimi 2015). The 6% reduction in accuracy in both images could be due to the avoided geolocation mismatch between land-cover map and reference data (usually from the field) and interpreter bias associated with the pixel count approach (Olofsson et al. 2014). The uncertainty estimates account for the sampling inconsistencies inherent in reference data collection. The overall accuracy of MLC for the 2015 image was lower than the 85% recommended by some scholars (e.g., Thomlinson et al. 1999; Ge et al. 2007; Foody 2008). The presence of high-density forest canopy over water surfaces contributed to high omission errors recorded for water in the 1986 map. Similarly, misclassifications of abandoned other tree-crop land, particularly citrus fields, which are overgrown and approaching secondary forest status, is probably the reason for high commission and omission errors. The combination of ISODATA and MLC classifiers reduced misclassification, which is a common flaw in mapping complex and heterogeneous landscapes from medium spatial resolution images such as Landsat. The aim of using two different classifiers was to harness the advantages of both classifiers. MLC is comparatively better than ISODATA and can even separate statistically inseparable land-cover types (Ahmad and Quegan 2013). The low accuracy value recorded is attributed to poor atmospheric conditions in the 2015 image and the fact that the reference and training data were collected a year after image capturing. In the face of a lack of independent training data for the historical image (1986), statistical clustering by ISODATA was an important initial step. The results obtained from the study show that ISODATA and MLC are suitable for landscape characterization. Recent literature (Coulter et al. 2016) suggests that the classification accuracies may be further enhanced by using vegetation indices, which could reduce the effect of atmosphere (clouds and haze).

### Changes in Landscape Composition

The main land-cover types making up the landscape—forest, food-crop land, cocoa and oil palm—remained the same between 1986 and 2015. However, the area has a high change to persistence ratio, meaning that the landscape is highly dynamic. Forests and food-crop land are declining, whereas the major tree crops (cocoa and oil palm) considerably increased in size. A minor increase in water bodies was also observed. The expansion of oil palm can be attributed to deliberate government policies to stimulate oil palm production, notably the President’s Special Initiative on oil palm of 2003 (Gilbert 2013) and the later tree-crop policy (MoFA 2012). The expansion of cocoa is explained by the suitability of a large part of the area for cocoa cultivation (Michel-Dounias et al. 2015) and the attractiveness of growing cocoa for farmers because of the status associated with having a cocoa farm, its guaranteed market at fixed prices, being a transferable property, and cocoa being a beneficiary of many government programs (subsidized seedling and fertilizer provision, mass spraying program, and agricultural extension services) (Laven 2010; Deans et al. 2017). The increasing visibility of water bodies is due to (i) deforestation along rivers as a result of which the water bodies are no longer hidden under vegetation cover, (ii) the presence of fish ponds, and (iii) the recent expansion of artisanal and small-scale gold and diamond mining (often illegal), which creates holes in which water is collected. Off-reserve forests have declined, but the persistence of contiguous forest in the center-north of the region is the result of its protected status as a forest reserve under the auspices of the Ghana Forestry Commission.

Change detection revealed a change in the geographical distribution of land-cover types resulting in increasing segregation of land uses and homogenization of the landscape. The mosaic landscape of 1986 transformed into one in which cocoa is being concentrated in the western part of the study area, oil palm in the eastern part, and a persistent component of contiguous forest remains in the central-northern part. Oil palm has taken over from cocoa in the eastern part of the study area. Explanations for these shifts include the relatively low-lying eastern side, which favors the growth of oil palm, but not cocoa, which prefers the drier areas in the west (Michel-Dounias et al. 2015). The proximity of the Ghana Oil Palm Development Company (GOPDC)—Ghana’s largest oil palm company—also has a role, both directly (it owns 5205 ha of oil palm plantations and 349 ha of smallholdings on the concession) and indirectly (about 13,000 ha of smallholders are in an outgrower arrangement with GOPDC) (http://www.gopdc-ltd.com/plantation/). Moreover, the proliferation of small- to medium-scale processing facilities provide a ready market for smallholders. Another pull factor for oil palm in the eastern part of the study area is the presence of the Oil Palm Research Institute, which facilitates the acquisition of improved seedlings and technology transfer, whereas the institute itself also has about 760 ha of oil palm plantations.

In addition to cocoa and oil palm dominance in the western and eastern areas, respectively, swaps have also been observed, meaning that some land-cover types have moved to another area, but retained their total size. They were high for cocoa, food-crop land, and oil palm (in declining order), making these three land-cover types key determinants of landscape dynamics. It has been suggested that this is part of the boom-and-bust cycle associated with cocoa in mosaic landscapes, with a “bust” occurring when yields decline, aged plantations are no longer regenerated and no remaining forest is available for further expansion (Clough et al. 2009). In Ghana, where remaining forests are confined to forest reserves where farming is not allowed, a new “boom” in a new “cocoa frontier” (Clough et al. 2009) is highly unlikely, unless existing farming land is massively converted to cocoa plantations. Only off-reserve forest patches can be—and are—cleared for cocoa establishment, with food crops as precursors or interplanted to provide food until canopy closure. After canopy closure, cocoa dominates, after which the old cocoa trees are felled and abandoned cocoa farms can be taken over by forest or are converted to oil palm (personal observation). Similarly, swamp forest is converted to oil palm, but rice is planted first. When oil palms become unproductive, they are either felled and the land converted to rice again, or left fallow and taken over by secondary forest. This results in cyclical movements of land cover alternating between forest, tree crops and food-crop land across the landscape. However, the rate of reverting land to forest is less than the conversion of forests as observed in western Ghana (Benefoh, personal communication).

### Intensity Analysis

Category intensity analysis showed, first, that forest is a dormant loser and gainer. Being a dormant loser is explained by the fact that a major part of the forest in the studied landscape is reserved under legal protection. The forest is a dormant gainer as a result of abandoned tree-crop plots being taken over by secondary forest, which barely occurs. Second, food-crop land is being converted to oil palm, cocoa, and settlements, at an intensity higher than overall category intensity. This transfer is systematic, meaning that gains in oil palm, cocoa and settlements target losses from food-crop land and implies increasing scarcity of land for food production. Third, cocoa and oil palm are active gainers and dormant losers at the landscape level, meaning that their expansion is above the average gains of all land-cover types in the landscape and, similarly, that their respective losses are below the average losses. Similar trends have been observed for cocoa in western Ghana (Benefoh, personal communication). This makes the two tree crops the dominant landscape components in the Akyemansa–Kwaebibrem landscape. Landscape dwellers associate tree-crop expansion and homogenization of the landscape with constrained ecosystem services and limited biodiversity, which is comparable with observations in Sulawesi (Belsky and Siebert 2003).

Transition intensity analysis revealed mainly that tree crops—cocoa and oil palm—replaced forests and food-crop land (Furumo and Aide 2017). With the majority of the remaining forest under protection and off-reserve forest reduced to small patches in usually inaccessible locations, food-crop areas are the most targeted for cocoa and oil palm expansion. These transitions may have serious implications for food security and forest and biodiversity conservation, as has been amply documented in the literature (Pfund et al. 2011; van Vliet et al. 2012; Castella et al. 2013). Transitions in cocoa and oil palm also targeted each other, meaning that cocoa was converted to oil palm and vice versa. However, in the study area the gains of oil palm from cocoa are larger than the gains of cocoa from oil palm. This means that oil palm is becoming a relatively stronger component in the landscape.

## Conclusions

The study addressed the question of how tree-crop expansion drives the spatial transitions in the mosaic landscape of a mixed cocoa/oil palm area in the Eastern Region of Ghana. It classified the mosaic landscape into land-cover types at two time points—1986 and 2015—to detect changes in landscape composition and processes behind the transformations. It revealed a dynamic landscape where tree-crop expansion is the main driver of landscape change, at the cost of off-reserve forests patches and food-crop land. Cocoa and forest reserves turned out to be stable components in the landscape, whereas oil palm is gradually expanding in absolute and relative terms. The analysis further showed that a previously mosaic landscape is in the process of increasing segregation and homogenization into mainly cocoa and oil palm. Commercial tree-crop cultivation has not only taken up 63% of the landscape, but also exhibits an expanding and aggregating trend. The findings imply risks of loss of biodiversity and other environmental services as well as decreasing food production potential. Landscape-level policies should target conservation of environmental services and agricultural production as twin objectives in landscape governance, for instance through effective land-use planning. Further research is required to quantitatively assess the configuration of the changing landscape as well as the effects of these changes on the provision of ecosystem services and peoples’ food security and livelihoods.

## Appendix

Table 7

**Table 7:** Original areas, adjusted areas and uncertainty of area estimates for the 1986 and 2015 land-cover maps

	Areas (km^2^) and confidence intervals (95%) for 1986 map			Areas (km^2^) and confidence intervals (95%) for 2015 map		
Land-cover categories	Original areas	Adjusted areas	Margin of error	Original areas	Adjusted area	Margin of error
Built-up/bare	30.35	30.96	± 0.98	33.75	34.32	± 0.54
Cocoa	280.01	250.95	± 6.27	381.26	304.74	± 5.57
Food crops	305.75	264.99	± 6.22	184.25	195.75	± 4.69
Oil palm	169.19	163.12	± 4.48	295.99	409.37	± 5.39
Other tree crops	22.04	38.25	± 3.29	40.99	44.73	± 2.31
Forest	326.74	376.30	± 5.52	191.6	139.43	± 4.38
Water	0.44	9.94	± 2.20	6.68	6.17	± 0.38
